# Enhanced conceptual understanding through formative assessment: results of a randomized controlled intervention study in physics classes

**DOI:** 10.1007/s11092-024-09445-6

**Published:** 2024-12-27

**Authors:** Andreas Lichtenberger, Sarah I. Hofer, Elsbeth Stern, Andreas Vaterlaus

**Affiliations:** 1 Laboratory for Solid State Physics, ETH Zurich, John-Von-Neumann-Weg 9, 8093 Zurich, Switzerland; 2 Chair of Education and Educational Psychology, Ludwig Maximilian University of Munich, Leopoldstrasse 13, 80802 Munich, Germany; 3 Chair for Research on Learning and Instruction, ETH Zurich, Clausiusstrasse 59, 8092 Zurich, Switzerland

**Keywords:** Formative assessment, Concept questions, Science education, Physics instruction

## Abstract

While formative assessment is a widely valued instructional approach to support meaningful learning, putting it into classroom practice remains a challenge, also because the time resources required may conflict with other goals. In a cluster-randomized controlled intervention study with 29 teachers and 604 students (mean age 15.6 years) at secondary school level, we examined the yield of formative assessment with regard to students’ conceptual understanding and quantitative problem-solving skills in physics. Ten teachers applied formative assessment (FA group) in a 14-lesson curriculum on kinematics after having undergone a training that focused on the implementation of multiple-choice concept questions together with monitoring tools, clicker sessions, and reflective lessons. In the frequent testing group (FT group), ten teachers had no training on formative assessment but implemented the same concept questions as those used in the FA group. Nine teachers taught kinematics in their traditional way (TT group). The results revealed that students in the FA group outperformed students in the other two groups in a test on conceptual understanding immediately after the intervention as well as 3 months later, whereas students from the FT group and the TT group did not differ. Importantly, a better conceptual understanding in the FA group was not at the expense of performance in quantitative problem solving, as students of this group better integrated both kinds of knowledge. Our study has shown that a short but well-structured formative assessment teacher training could unfold its potential in terms of students’ learning of challenging content.

**Supplementary Information:**

The online version contains supplementary material available at 10.1007/s11092-024-09445-6.

## Introduction

Over the past decades, researchers, education policy-makers, and teachers have become aware of the constructivist nature of learning, which means that students process incoming information by connecting it with their existing knowledge (Scardamalia & Bereiter, 2006; Staub & Stern, 2002). Since students differ in their prior knowledge, they also vary in how they represent, understand, and interpret the presented content. Prior knowledge often makes learning easier, but it can sometimes be an obstacle. This is the case when a student has already developed conceptual frameworks that work well under certain circumstances but differ from or even contradict scientific explanations. So-called alternative concepts—sometimes also labeled as misconceptions—can be found in all content areas, but are particularly widespread in science, especially in physics (Hake, 1998; Hestenes et al., 1992; Hofer et al., 2018; Lasry et al., 2014; Schecker et al., 2018). It is widely agreed that carefully prepared study materials and well-prepared lessons are doomed to failure if teachers do not adequately take into account the students’ prior knowledge (Carey, 2000). Insights into the pivotal role of prior knowledge for future learning raised awareness for the potential of using assessments not only to evaluate students’ learning at the end of an instructional unit (summative assessment, assessment *of* learning), but also for monitoring students’ learning during instruction to provide ongoing adaptive feedback (formative assessment, assessment *for* learning). According to Black and Wiliam (1998, 2009), formative assessment can be understood as a unified method of regularly applying assessments to gather evidence of student learning. The evidence is then interpreted and used by teachers to design and adapt classroom activities to their students’ needs and benefits. William and Thompson (2008) proposed the following five key strategies that operationalize formative assessment and should make it effective for implementing in practice: (1) clarifying, understanding, and sharing learning intentions and criteria for success; (2) engineering effective classroom discussions and other learning tasks that elicit evidence of student understanding; (3) providing feedback that moves learners forward; (4) activating students as instructional resources for one another; and (5) activating students as the owners of their own learning. These strategies are still state of the art (Cizek et al., 2019; Wiliam, 2019) and have been applied to learners at various age levels and school types and across a broad range of content areas and subjects (Andrade et al., 2019), specifically also to mathematics classes (e.g., Andersson & Palm, 2017; Boström & Palm, 2023; Rakoczy et al., 2019) and science classes (Shavelson et al., 2008) at both primary and secondary school level. While few educators and researchers dispute the potential of formative assessment, there is consensus that further research is necessary to determine the size of its effect on achievement across different student populations and contents (e.g., Bennett, 2011; Dunn & Mulvenon, 2009). Furthermore, existing studies are often difficult to compare, as the formative assessment implemented by teachers was either not described carefully enough or employed different strategies. Studies on formative assessment with large, randomized samples, control groups, and a detailed description of how the strategies of formative assessment were implemented are scarce (Boström & Palm, 2023; Schneider & Randel, 2010).

We implemented a formative assessment program that employed concept questions based on the five key strategies of William and Thompson (2008) within a 14-lesson physics curriculum on kinematics. The effectiveness of this program was investigated in a cluster-randomized controlled intervention study with students from gymnasium schools (an advanced secondary school that allows admission to university). These students most likely score at the upper part of the cognitive ability scale and have demonstrated themselves to be efficient and self-regulated learners. Hence, they may recognize their deficits on their own and improve their conceptual understanding when being faced with concept questions, also without the explicit application of formative assessment. We thus wanted to determine whether and to what extent there is an effect of formative assessment beyond the presentation of conceptual problems.

Learning physics, and kinematics in particular, is difficult even for otherwise capable students. Reaching the highest level of competence requires integrating different external representations, that is, conceptual explanations, formulae, graphs, tables, and outcomes of experiments (Angell et al., 2004). Traditional teaching in physics has a strong emphasis on practicing quantitative problems, and students are likely to improve in applying the correct formulae (Kim & Pak, 2002; Taconis et al., 2001). There is, however, overwhelming evidence from research on physics education at the high school and college levels that the correct application of formulae does not automatically go along with conceptual understanding. A substantial number of students who succeed in solving quantitative problems have not acquired a proper conceptual understanding (Clement, 1982; Gunstone, 1986; Hake, 1998; Halloun & Hestenes, 1985; Hestenes et al., 1992; Hofer et al., 2018; Mazur, 1997a; McDermott, 1984; Planinic et al., 2012). As our implementation of formative assessment focuses on conceptual understanding, less time could be spent on learning quantitative problem-solving procedures. Besides the effectiveness of formative assessment regarding conceptual understanding, we therefore also investigated whether, and to what extent, formative assessment with a focus on conceptual understanding comes at the expense of quantitative problem solving.

## Challenges of implementing formative assessment in physics classes

### Putting formative assessment into practice

Educational researchers agree that teachers should not understand formative assessment as a recipe to be used as directed. They rather should have internalized the idea of the constructive nature of learning, which contrasts with the outdated notion of learners as empty vessels to be filled with carefully prepared pieces of knowledge. However, given the complexity of classroom practice, developing and implementing means of formative assessment remains challenging.

Andrade et al. (2019) agree that formative assessment involves both domain-specific and domain-general components. In physics education, formative assessment usually goes along with implementing appropriate conceptual problems (Furtak et al., 2019). To select and apply such problems for the assessment procedure, one must understand the core content to be taught and anticipate students’ specific difficulties in mastering the learning goals. This demands both content knowledge and pedagogical content knowledge in the sense of Shulman (1986, 1987) and concerns the domain-specific component. Furtak et al. (2016), who investigated biology teacher’s development of formative assessment abilities in a 4-year intervention study, revealed that after 3 years, almost all teachers improved considerably in interpreting students’ ideas and in providing helpful feedback. Less consistent, however, was teachers’ progress in designing formative assessment tasks of high quality. This is not too surprising, given the effort that is needed to create and select questions that depict conceptual understanding.

Independent of the content area and the subject to be taught, there are common domain-general elements behind the mission of formative assessment, which are reflected in the five key strategies by William and Thompson (2008) mentioned in the beginning. A challenge for teachers is that the implementation of these strategies requires them to act as a facilitator of learning rather than as an instructor, a role shift that is likely to unsettle even teachers convinced of formative assessment. Johnson et al. (2019) revealed that many in-service teachers who had undergone trainings on formative assessment had still difficulties with supporting students in taking control of their own learning by giving them the opportunity to figure out their misunderstandings for themselves. Teachers do not seem entirely convinced that students will use time for meaningful learning activities without direct guidance.

Given the effort required to get the full formative assessment program work, a lighter version that focuses on diagnosing misunderstandings might serve its purpose. Research in physics education has shown that presenting students with conceptual questions can already promote conceptual understanding, even when not all strategies are implemented (Mazur, 1997b; Molin et al., 2021). Additionally, there is evidence of test-enhanced learning when students are repeatedly presented with similar questions (McDaniel et al., 2007). Therefore, it seems worthwhile for studies on formative assessment to decouple the effect of frequently applying conceptual questions (domain-specific) from the effects of implementing the key strategies of formative assessment (domain-general).

### Mastering kinematics

**Fig. 1:**
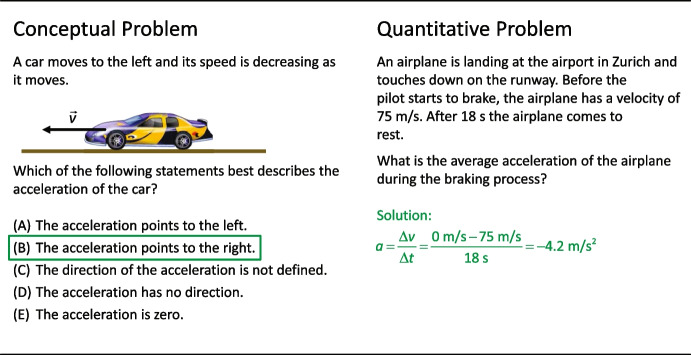
A conceptual and a quantitative problem about kinematics. Solving the conceptual question on the left requires deeper understanding of the concept of acceleration. To solve the quantitative problem on the right, a mathematical procedure must be applied

Figure 1 juxtaposes two problems from kinematics about an object that slows down. Although both problems deal with the acceleration of the object, they are different in nature. The one on the left side is a conceptual problem that requires a deeper understanding of acceleration within the theoretical framework of kinematics. The one on the right side is a quantitative problem that requires the application of a formula that models the relationship between the variables.

A main reason for difficulties related to conceptual understanding in physics is that scientific concepts often differ from, or even contradict, students’ naïve beliefs and explanations derived from their experience in interacting with the physical world (Carey, 2000; Savion, 2009). Hence, students enter classes with beliefs and concepts that are likely to interfere with the scientifically accepted explanations provided by teachers. This can lead them to ignore their teacher’s explanations or construct new synthetic concepts that do not coincide with scientific models (Mazur, 2015; Vosniadou et al., 2008). Naïve notions about concepts from kinematics, such as the magnitude and direction of velocity and acceleration, may be particularly resistant to change since the motion of objects is a natural part of one’s everyday experience from early on. Even preschool children have an implicit understanding of the trajectories of moving objects when playing with balls or other toys (Wilkening & Cacchione, 2010). When confronted with kinematics instruction at school, however, students are likely to be confused. They hear familiar words such as movement, motion, speed, or acceleration, for which they have created meanings and images that differ from the scientific view. For example, Rosenquist and McDermott (1987) showed that many students have difficulties distinguishing between the position and velocity of objects. Specifically, they found that students often mistakenly believe that during an overtaking maneuver, both objects are moving at the same speed once they are in the same position. A similar challenge concerns the distinction between velocity and acceleration, which is typically grounded in everyday experience, as illustrated in the following example. If a child hits a toy with wheels on a flat floor, it will roll for a while. The same happens if they release the toy on a ramp. It is far from plausible that in the first case, a negative, and in the second case, a positive acceleration is acting during the toy’s free movement, as both objects move forward. The directional aspect of acceleration typically causes additional difficulties. Depending on the frame of reference, a “positive” acceleration may refer either to an increasing or a decreasing speed. The same holds for “negative” acceleration. If a car drives in the opposite direction and is therefore labeled as traveling in a negative direction, a positive acceleration will decrease its speed, while a negative acceleration will make it move faster. The ambiguity of the meaning of “positive” and “negative” contradicts the everyday use of these words, where “positive” is linked to growth and “negative” is linked to reduction. These examples illustrate that there are good reasons to believe that the transition from an everyday understanding to a scientific understanding of acceleration requires a radical conceptual restructuring rather than just an accretion of new facts in the sense of Carey (1985). The need for knowledge restructuring applies to all concepts of kinematics, as many studies about student beliefs in that content area have shown (Beichner, 1994; McDermott et al., 1987; Planinic et al., 2013).

While several studies have shown that students improve their conceptual understanding when they get opportunities to become aware of their naïve beliefs and compare them with scientific explanations (Crouch & Mazur, 2001; Hake, 1998; Hofer et al., 2018; Ploetzner et al., 1999), this knowledge restructuring takes time and may come at the expense of quantitative problem solving. Being competent in mathematics and formal sciences like physics involves not only understanding concepts but also mastering formal procedures, which is typically the focus of traditional instruction (Mazur, 2015) and can be efficiently promoted by presenting the students with worked-out examples and exercises (Renkl, 2014). For elementary school mathematics, it has in fact been shown that formative assessment improved conceptual understanding at the expense of numerical procedures (Clarke et al., 2014). There are reasons to believe that this is not the case at higher school levels, as advanced learners most likely have a sufficient command of the necessary mathematical procedures but struggle with mapping them to concepts. Several studies in physics have shown that dedicating class time to conceptual restructuring and understanding is valuable and does not come at the expense of solving quantitative problems (Crouch & Mazur, 2001; Hofer et al., 2018; Ploetzner et al., 1999). Once a deeper conceptual understanding has been reached, formulae can be integrated into the knowledge representations even without extensive practice. In contrast, practicing quantitative problem solving rarely supports the acquisition of conceptual knowledge (Kim & Pak, 2002; Taconis et al., 2001). Given the conceptual hurdles in understanding kinematics, the content seems a worthwhile example for investigating the potential benefits of formative assessment in conceptual understanding, while controlling for potential detriments in quantitative problem solving.

## The present study: design, research questions, and hypotheses

We developed a formative assessment program including four tools based on the five key strategies suggested by William and Thompson (2008), which can be embedded in a kinematics curriculum and directly put into practice. They comprise multiple-choice questions, which are implemented together with peer discussion (so-called clicker sessions), a diagnostic multiple-choice test with distractors (incorrect answers) derived from learners’ statements in interviews, a monitoring tool to track the learning process, and a reflective lesson to make up for the diagnosed deficits (a more detailed description follows in the “Materials” section). Our design took into account some obstacles in putting formative assessment into practice that have been highlighted in the research of the past years. First, we support teachers by designing a set of appropriate concept questions, which can be directly used for formative assessment. Second, to mitigate teachers’ concerns about the role change from instructor to facilitator, our formative assessment program puts a strong focus on ready-to-use monitoring and feedback tools embedded into the curriculum (Decristan et al., 2015). For example, during the clicker sessions, we use an electronic classroom response system to structure peer and classroom activities. Furthermore, we created the reflective lesson where teachers are given feedback and materials that facilitate them to put the responsibility of learning in students’ hands. These approaches are likely to protect teachers from feelings of loss of control and help students become aware of what they need to work on.

Students learned about seven basic kinematics concepts, which are already extensively described in the literature (Beichner, 1994; McDermott et al., 1987; Planinic et al., 2013): velocity as rate, velocity as a one- and two-dimensional vector, displacement as the area under the velocity*–*time curve, acceleration as rate, acceleration as a one-dimensional vector, and velocity change as the area under the acceleration–time curve.

To disentangle the impact of providing teachers with concept questions from the impact of embedding them in a formative assessment program using the five key strategies by William and Thompson (2008), we developed a second program with a mere focus on applying the same concept questions used in the full formative assessment program.

Since the formative assessment program employs four tools focused on conceptual understanding, leaving less time for practicing quantitative problems, we aimed to test whether this focus negatively affects numerical problem-solving skills.

Based on these considerations, we randomly assigned the in-service teachers, who gave consent to participate in the study, into three groups:

1. Formative assessment group (FA group): Teachers received comprehensive training on how to implement multiple-choice problems for formative assessment and how to adapt them for classroom practice (for details see the “Methods” section).
2. Frequent testing group (FT group): Teachers were asked to present their students with the same conceptual problems as those used in the FA group, but were not given training on formative assessment.
3. Traditional teaching group (TT group): Teachers were asked to teach kinematics in their traditional way.

Using a pretest, posttest, and follow-up test on conceptual understanding and a posttest on quantitative problem solving, comparisons between the groups allowed us to address the following research questions: (1) Does a comprehensive implementation model of formative assessment show stronger effects on students’ conceptual understanding than the mere presentation of conceptual problems? If the five key strategies of formative assessment unfold their full potential in classes of advanced learners, we expected students of the FA group to outperform students of the FT and TT groups (Hypothesis 1). (2) Does the mere use of concept questions already improve students’ conceptual understanding? If advanced learners benefit from being presented with conceptual problems, we expected the students of the FT group to outperform students of the TT group (Hypothesis 2). (3) Is the focus on conceptual understanding at the expense of quantitative problem-solving skills? If improved conceptual understanding supports integrating mathematical modeling, in line with the mentioned findings in physics, we expected that the FA group could compensate for the reduced time spent practicing those skills, and therefore would not be outperformed by the TT and FT groups in the quantitative problem-solving test (Hypothesis 3a). In addition to comparing mean scores across the three groups, we also compared the correlations between conceptual understanding and quantitative problem-solving skills, which reflect the degree to which formulae and concepts are integrated. We expected the correlation to be lower in the TT group than in the other two groups (Hypothesis 3b).

Stimulated by previous findings, we also exploratively investigated the impact of formative assessment on promoting female students. Latest in secondary school, gender imbalance in physics becomes evident: male students show a higher degree of interest in the subject and outperform their female counterparts (Cimpian et al., 2020; Halpern, 2014; Hofer & Stern, 2016). Among the various attempts to narrow the gender gap, a stronger focus on conceptual understanding was promising (Hofer et al., 2018; Lorenzo et al., 2006). Based on these findings we were interested in whether the gender gap would be smallest in the FA group.

## Methods

### Participants

A total of 29 teachers from 25 gymnasiums in the German-speaking part of Switzerland participated in our cluster-randomized field study. The gymnasium is a type of public school in Central Europe that provides higher secondary education for above-average achieving students, comparable to U.S. high school students attending college preparatory classes. In Switzerland, approximately 20% of a cohort attends a gymnasium, and a degree from a gymnasium enables admission to all universities. The 29 gymnasium teachers (25 male, 4 female) were recruited on a voluntary basis from a professional development program. In the first step, the teachers were informed about the design and the planned random assignment. The teachers not assigned to the FA group were promised the training after the end of the study. The teachers participated with one or two of their regular classes. The mean class size was 20 students (SD = 3), with a range from 14 to 25 students. In Switzerland, all gymnasium teachers need a master’s degree in their subject (in our case, physics) and a degree from an academic teacher education program.

From the classes of the 29 teachers, 704 students participated in the study. However, only the 628 students who completed the pretest and posttest on conceptual understanding and the posttest on quantitative problem solving in kinematics were included in our analysis. Twenty-four students were excluded because they only clicked through the tests without seriously answering the problems (as indicated by an evaluation of click response times). Finally, we checked the data sets for test-specific outliers. To identify outliers, we used the three-sigma rule of thumb (Smirnov & Dunin-Barkowski, 1963). Considering the posttest on conceptual understanding, we found three scores that differed by more than three standard deviations of the mean. In the conventional test, we did not find extreme scores. All scores lied within 2.4 standard deviations of the mean in the respective groups. Thus, the main analysis was conducted with a data set of 604 students (no missing values). The follow-up analysis 3 months later was conducted with a data set of 561 students. Thus, we had an attrition rate of about 8% between posttest and follow-up test, which is attributed to only little bias.

After the random assignment of teachers to the groups, we had 9 teachers with 12 classes and 215 students in the TT group (59% female, mean age *M* = 15.5 years, *SD* = 1.1 years), 10 teachers with 12 classes and 207 students in the FT group (47% female, mean age *M* = 15.6 years, *SD* = 0.8 years), and 10 teachers with 11 classes and 182 students in the FA group (53% female, mean age *M* = 15.6 years, *SD* = 0.8 years). The different number of students per teacher and the varying gender proportions across groups were taken into account in the analyses.

All participants (resp. their parents) gave written consent to participate in the study. All collected data was completely anonymized. We followed the ethical guidelines of our university’s Ethics Commission who approved the study.

We conducted a Monte Carlo simulation in Mplus to evaluate the statistical power of our analysis model. Based on the lower bound of effect sizes found in prior formative assessment studies (e.g., Andersson & Palm, 2017; Bell & Cowie, 2001; Black & Wiliam, 2009; Shepard, 2000), we specified an effect size of Cohen's *d* = 0.4 for the effects of the intervention condition in comparison to the control condition. The power analysis showed that with our planned design and a target sample size of 30 teachers with approximately 600 students, we achieved an adequate power of 0.84.

### Procedure

**Fig. 2:**
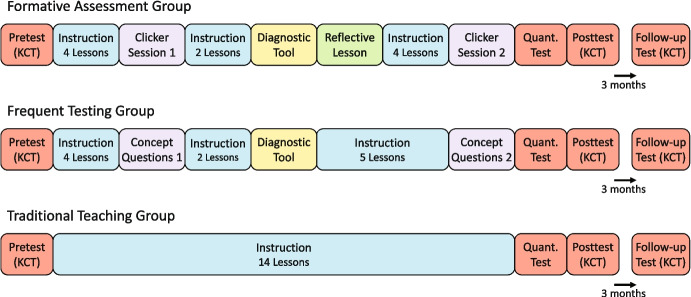
Procedure for each of the three groups. Teachers in the traditional teaching group delivered 14 lessons on kinematics in their usual way. In contrast, teachers in the frequent testing group incorporated concept questions in three predefined lessons, while teachers in the formative assessment groups implemented formative assessment tools—specifically clicker sessions, a diagnostic tool, and a reflective lesson—in four designated lessons. A test on conceptual understanding was applied directly before, immediately after, and 3 months after the intervention. Additionally, a quantitative problem-solving test was administered after the intervention

To examine our research questions, we implemented a cluster-randomized controlled intervention with three groups (FA, FT, and TT) in real classroom conditions. All groups participated in 14 lessons of kinematics instruction. Figure 2 illustrates commonalities and differences among the procedures in the three groups. To assess learning, we administered a test on conceptual understanding as pretest, posttest, and follow-up test and a test on quantitative problemsolving as posttest. The different lessons depicted in Fig. 2 (e.g., “Reflective Lesson”) will be explained in the “Materials” section.

Before the classroom intervention in the classroom started, we organized a half-day teacher training for all teachers where we explained the general strategy of the project, what formative assessment is, and how the teachers had to document their instructions. To implement our design appropriately, we had to ensure that the same topics were taught in all classes and that the lessons assigned to the topics were the same in all classes. Therefore, all the teachers were told that the kinematics curriculum should cover the seven basic kinematics concepts mentioned above and that they should assign 14 lessons to the topic. The students were not given any homework during this time, so the amount of time spent on the study materials did not vary between the classes. All teachers were asked to teach the topics in their usual ways, but the teachers assigned to the FT group were asked to reserve three lessons to implement the concept questions, and the teachers of the FA group were asked to set aside four lessons to implement the four tools along with the formative assessment strategies.

The teachers assigned to the FA group stayed for training on formative assessment. We explained the four tools we developed to implement formative assessment. Each teacher was provided with a class response system (Turning Point by Turning Technologies) and a laptop with the required software for conducting clicker sessions preinstalled. The teachers were then instructed on the exact procedure for using multiple-choice concept questions in clicker session and had the opportunity to practice it. They also received a class set of monitoring tool booklets and were told how students should use them. Lastly, we explained how to design a reflective lesson based on the feedback from the diagnostic test. The formative assessment elements could easily be embedded into the standard curriculum. The teachers only had to ensure that the concepts covered in the clicker sessions were taught beforehand. Apart from that, they were free to develop and use their own instructional materials.

The teachers assigned to the FT group were instructed on implementing the same set of multiple-choice concept questions as those used in the FA group. Instead of being involved in clicker sessions, students in the FT group solved the clicker questions on a computer during a lesson, and feedback was limited to showing them the correct answers. The diagnostic test was applied to the FT group in the same way as to the FA group. However, neither students nor teachers received feedback from the diagnostic test, and no reflective lesson was included. It was left to the teachers to determine how to use the information from the testing in the classroom.

It is worth noting that we worked against ourselves with regard to this implementation procedure because the TT group teachers had a better chance of applying their regular classroom practice than did the teachers of the other two groups.

## Materials

We first explain the implementation of the formative assessment in the FA group. We then discuss the multiple-choice concept questions used in the FA and FT groups and present some more examples. Finally, we describe the measures applied in the study.

### Implementation of formative assessment in the FA group

We developed four tools to implement the five key strategies (see Table 1) of formative assessment defined by Black and Wiliam (1998): clicker sessions, a monitoring tool, a diagnostic test, and a reflective lesson. Table 1 shows how these tools were embedded into the schema by William and Thompson (2008). In what follows, we briefly describe the tools, their implementation, and their aims.

**Table 1:** Key strategies of formative assessment and how we implemented them by four means: clicker sessions, a monitoring tool, a diagnostic test, and a reflective lesson

	Where the learner is going	Where the learner is right now	How to get there
Teacher	**1** Clarifying learning intentions and criteria for success *Explicit definition of the concepts and criteria for success in the ****monitoring tool***	**2** Engineering effective classroom discussions and other learning tasks that elicit evidence of student understanding ***Clicker sessions**** and their evaluation by means of the ****monitoring tool***	**3** Providing feedback that moves learners forward *Feedback about conceptual knowledge and prevalent misconceptions from a ****diagnostic test**** followed by a ****reflective lesson***
Peer	Understanding and sharing learning intentions and criteria for success *Supporting peers with the ****monitoring tool***	**4** Activating students as instructional resources for one another *Peer instruction during the**** clicker sessions***	
Learner	Understanding learning intentions and criteria for success *Working with the ****monitoring tool***	**5** Activating students as the owners of their own learning *Self-responsibility in selecting the learning material for the ****reflective lesson***	

Key strategies are from William and Thompson (2008). Our implementation is described in italics

#### Clicker sessions

**Fig. 3:**
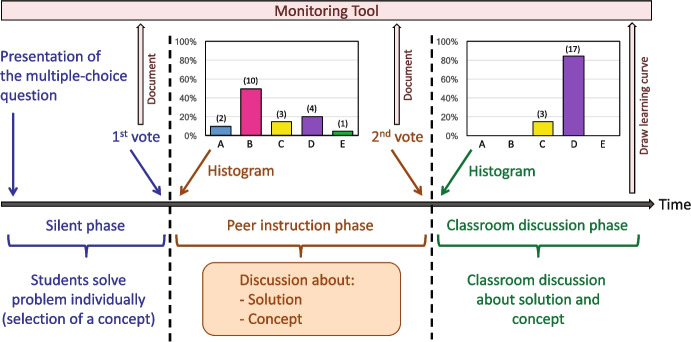
Diagram of the procedure of a multiple-choice concept question during a clicker session. Three phases can be distinguished: the silent phase where students answer the question alone, the peer discussion phase where students discuss the problem in small groups, and the classroom discussion phase where the teacher discusses the correct answer and the distractors in class

To make students’ thinking visible (key strategy 2, Table 1), we designed classroom activities called clicker sessions. A clicker session consists of a sequence of concept questions in a multiple-choice format with one correct answer and distractors based on common misconceptions. The implementation of the questions follows a strict procedure adapted from Mazur’s peer instruction (Bruff, 2009; Mazur, 1997a), which is illustrated in Fig. 3. Each question is projected on the wall, and students first answer the question on their own. Moreover, the students must assign a concept, or several if necessary, to the question. This is to prevent them from guessing the answers and to make them aware that problems can be solved more effectively by using physics concepts rather than by relying on their gut feeling (Maries & Singh, 2013). Using a handheld electronic response device, the students submit their answers anonymously, and a histogram of the votes is shown to the entire class. In the next step, the students discuss their answers in groups of three and are encouraged to convince each other of their selected answers. This approach allows the students to learn from their peers’ thought processes, and they are activated as instructional resources in a natural way (key strategy 4, Table 1). After the peer discussion, the students vote a second time, and a histogram with all responses is presented to the class, highlighting the correct answer. In an interactive classroom discussion, the teacher explains why the indicated answer is correct and why the distractors are incorrect. In the clicker sessions, students receive feedback on the accuracy of their conceptual knowledge, and teachers learn from the histograms the proportion of students with correct and incorrect beliefs.

#### Monitoring tool

**Fig. 4:**
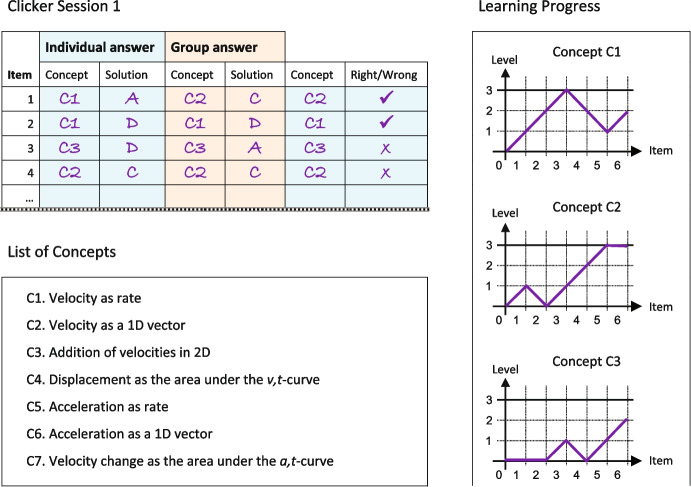
Excerpt of the monitoring tool where students record their answers to concept questions during the clicker session. During the clicker session, the students write their answers and the concepts their answers are based on in the corresponding fields of the table. After the classroom discussion, they document the correct assignment of concepts to the questions and whether their own answers were right or wrong in the last two columns. The seven concepts are listed so that students can use them as a resource for their answers. Finally, the students transfer the results of the last two columns into the learning progress diagram for each concept

During a clicker session, students use a paper-based monitoring tool to track their learning progress toward the learning goals. The booklet contains a list of all the concepts students have to learn, a table where they fill in their answers during the clicker session, and diagrams used to record and visualize the learning trajectory over time. Figure 4 shows an excerpt of the monitoring tool. The learning curve in the diagram is constructed as follows: If the answer is correct, the level increases by one, and if it is incorrect, the level decreases by one. The decision of whether an answer is right or wrong is the responsibility of the students. For example, if a wrong answer is chosen by mistake, they are allowed to rate it as correct. We have introduced two limiters. The lower limiter is set to zero so that the level never goes negative. Students who have difficulties at the beginning of the session still have the possibility to reach the top level in the end. The upper limiter is set to three. Thus, students who initially give correct answers but later become confused by the interference of other concepts (Sayre & Heckler, 2009; Scaife & Heckler, 2011) will quickly return to the zero level. This is supposed to help them realize that the initial concept was not properly understood. The tool urges students to analyze their learning progress by asking questions such as “Where do I want to go?” along with the list of the leaning goals. Students can identify the difference between their actual knowledge state and the learning goals. Hence, the monitoring tool serves the implementation of the first and second key strategies in Table 1.

#### Diagnostic test

Students already receive feedback about their performance during the clicker sessions when discussing their ideas with peers and when comparing their own answers to the correct solutions. However, only a limited number of questions can be discussed within a clicker session, and the decision of whether a question is answered correctly or not lies in the hands of the students. To provide them with more detailed and objective feedback about their conceptual knowledge, we designed a test with 33 multiple-choice concept questions to be administered after instruction on the four concepts about velocity. Every question in the test can be assigned to a single concept, and every distractor can be associated with a common misconception (an example is given in the next section). After completing the diagnostic test on a computer, students immediately obtain their results by e-mail. The feedback includes a bar plot for overall performance, a radar plot illustrating percentages of correct answers per concept, and a second bar plot showing the presence of misconceptions. The teacher receives the same plots for the class averages. By highlighting not only students’ knowledge of the concepts but also the misconceptions still present, the diagnostic test helps both students and teachers address the question, “What kind of knowledge is needed to reach the goal?” This takes into account key strategy 3 in Table 1, according to which learners must be given the opportunity to work on their weaknesses and actively reduce their misconceptions.

#### Reflective lesson

As part of the lesson preparation, teachers are asked to analyze the results of the diagnostic test and design materials that meet the needs of different students. During the lesson, depending on the outcome of the diagnostic test, the students themselves decide which learning goals and materials they want to work on. Furthermore, the students are encouraged to compare the results of the diagnostic test with the learning progress documented in the monitoring tool. A discrepancy (e.g., a high level in the monitoring tool and a low level in the diagnostic test) should encourage further observations during the reflective lesson. By giving them responsibility and prompting reflections on their learning, the reflective lesson is supposed to support students on their way to becoming self-directed learners (key strategy 5, Table 1). For the reflective lesson, we developed a set of problems for each concept to help teachers choose those that meet the needs of their students.

### Concept questions used in the FA and FT group

**Fig. 5:**
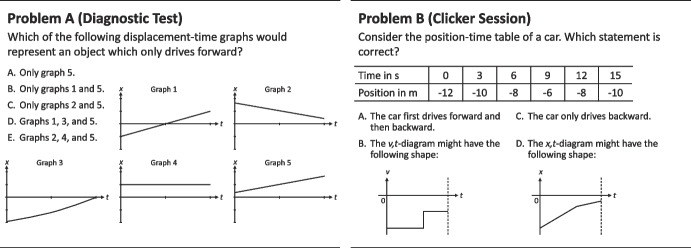
Concept problems about kinematics from the diagnostic test and a clicker session. Problem A from the diagnostic test uses only graphs (single representation) and can be clearly assigned to a single concept, namely the direction of velocity. In contrast, Problem B from the clicker session includes tables, texts, and graphs (multiple representations) and can be associated with different concepts, such as the direction of velocity and the rate of velocity

The multiple-choice questions used in the diagnostic test and in the clicker sessions had different functions and therefore differed in their design. Figure 5 presents two examples, one from the diagnostic test and one from a clicker session. Both problems require conceptual knowledge about the direction of velocity.

Problems used in the diagnostic test are assigned to exactly one kinematics concept and one representation. Furthermore, distractors are chosen to target common misconceptions. This allows teachers and students to obtain a fine-grained analysis of the current knowledge and to trace back mistakes to specific misconceptions. A typical misconception is expressed in answer C to Problem A in Fig. 5, where a positive direction is associated with a position with positive coordinates.

Problems used in the clicker session are designed to stimulate learning processes during peer discussions. They can involve more than one concept and representation and are supposed to encourage students to compare and contrast concepts and representations during their interaction. For example, in Problem B in Fig. 5 (right-hand side), different representations are used for the problem (table) and for the distractors (text/graph). During the clicker session, students are told to assign the dominant concept in solving the question. This can differ between students in some cases as some problems might be solved by applying different concepts. The teacher can take up students’ suggestions in the classroom discussion.

### Measures

#### Students’ personal characteristics

One week before the start of the intervention, the students received a link to an online questionnaire where they were asked to report their age and gender.

#### Students’ conceptual understanding

We used the validated Kinematics Concept Test (KCT, Lichtenberger et al., 2017) to measure the students’ conceptual understanding. This test consists of 49 multiple-choice items (resulting in a maximum score of 49) with a single correct answer and three to five distractors. Each item can be assigned to one of the seven kinematics concepts covered during instruction (for more details, see Lichtenberger et al., 2017). An exemplary item of the KCT is shown in Fig. 1 (left-hand side). The test was presented as pretest, posttest, and follow-up test (3 months later) on an online platform with a code to open the test. Test access was closed immediately upon completion. The teachers did not know the items used in the KCT. The reliabilities (Cronbach’s *α*) for the KCT were similar at all measurement points (pretest: *α* = 0.88; posttest: *α* = 0.91; follow-up test: *α* = 0.92). Between-group deviations were negligible (differences < 0.02).

#### Students’ quantitative problem-solving skills

Traditional instruction on kinematics has a strong focus on modeling the quantitative relationships between variables, as in the exemplary problem in Fig. 1 (right-hand side). To assess these quantitative problem-solving skills, we composed a test consisting of five standard problems from textbooks that require intermediate steps and therefore allow for a graduated evaluation. The problems appear in contexts slightly different from those used in the textbooks to make sure that none of the students had solved one of the problems already during instruction. Points are awarded for correct steps in the solving process, e.g., for correct formulae, intermediate results, and final calculations. The resulting maximum score for a correct solution to all five problems is 35. Thirteen experienced Swiss gymnasium teachers confirmed that the selected problems adequately covered the contents. The test was presented at the end of the teaching unit. It was sent as a paper–pencil version to the teachers only 1 week before they applied it to prevent teaching to the test. A reliability of *α* = 0.70 was achieved, with negligible between-group differences. An English translation of the test, along with the assignment of problems to the concepts, the solutions, and the rating grid, is available in Supplementary File 1 accompanying the article.

#### Students’ cognitive ability

To control for the impact of intelligence on learning kinematics, the students were presented with Set II of the Advanced Progressive Matrices Test (Raven et al., 1992) 1 week before the start of the intervention. This test is one of the most commonly used nonverbal intelligence tests, with a maximum score of 36. The reliability of *α* = 0.75 was slightly lower than the reliability reported in the manual (*α* = 0.84), which can be traced back to the selected sample of gymnasium students, but it is considered high enough to use the test as a predictor variable. The test was administered by a member of our study team following the instructions in the manual.

#### Teachers’ pedagogical content knowledge

Although the 29 teachers were randomly assigned to the three groups, we additionally controlled for a selection bias at the teacher level by presenting a questionnaire on pedagogical content knowledge (PCK) in physics. The questionnaire with a maximum score of 24 was developed by Kirschner et al. (2016). It follows the conceptualization of Shulman (1987), who understands PCK as “the blending of content and pedagogy into an understanding of how particular problems, topics or issues are organized, represented and adapted to the diverse interests and abilities of learners, and presented for instruction” (p. 8). The questionnaire comprises twelve questions dealing with the interplay of subject-specific knowledge and pedagogy related to the instruction of mechanics and electricity. For example, teachers are required to identify typical student misconceptions, to rate students’ conceptual explanations, to name pedagogical and subject-related functions of instructional strategies, and to evaluate the extent to which certain teaching sequences contribute to meaningful learning.

## Implementation fidelity

To control for implementation fidelity of the formative assessment, we visited all clicker sessions in the FA group. During these sessions, the teachers applied a total of 133 conceptual problems. Analysis of the monitoring protocols showed that the teachers mostly adhered to the instructions. The first phase, in which the students answered the clicker questions individually, was implemented correctly in all cases. The subsequent group discussions were conducted correctly for 126 problems (95%). Concluding classroom discussions were also largely implemented correctly. They were skipped in ten instances where all the students had already chosen the correct answer after the peer discussion. In an additional seven cases, the discussion was omitted due to lack of time. Evaluation of the teaching materials showed that all teachers had taught the concepts covered in the clicker sessions beforehand. We also audio-recorded the students’ peer discussions during the clicker sessions and collected their monitoring tool booklets. In this way, we could compare their personal records to the answers they submitted via the classroom response system. To check the implementation of the reflective lesson, we collected and inspected the materials applied by the teachers.

All the teachers participating in the study wrote reports about their lessons (including contents, methods, and forms of teaching) and handed in their complete teaching materials. To validate their protocols, we also visited and monitored two lessons from each teacher in both the FT and TT groups. Our analyses indicated high implementation fidelity for both the FA and FT interventions, and also for the behavior of the teachers in the TT group.

## Results

The descriptive statistics of the students’ achievement are depicted in Table 2.

**Table 2:** Group-specific means and standard deviations for the scores of the KCT pretest, posttest, follow-up test, and quantitative test

	TT group				FT group				FA group		
	*M*	*SD*	*N*		*M*	*SD*	*N*		*M*	*SD*	*N*
KCT pre	15.4	7.8	215		16.2	8.3	207		16.8	8.2	182
KCT post	24.3	9.4	215		27.0	9.7	207		30.5	8.9	182
KCT follow-up	25.1	9.7	206		27.4	9.8	185		31.2	9.8	170
Quantitative test post	18.2	7.6	215		16.4	7.8	207		17.3	8.1	182

In the first step, we checked for selection bias at the teacher and student levels. At the teacher level, we considered the scores of the questionnaire on pedagogical content knowledge (TT: *M* = 16.1, *SD* = 3.1; FT: *M* = 16.2, *SD* = 2.4; FA: *M* = 17.1, *SD* = 3.2) in a one-way analysis, which showed no significant differences; *p* > 0.20. At the student level, one-way analyses were run with the raw scores from the Raven test (TT: *M* = 25.9, *SD* = 4.6; FT: *M* = 25.8, *SD* = 5.0; FA: *M* = 26.5, *SD* = 4.1) and the scores from the KCT pretest (Table 2). No between-group differences were found (*p* > 0.20).

In our design, the students were clustered in classes with different teachers. We therefore applied hierarchical linear modeling (Hox, 2010; Raudenbush & Bryk, 2002) to analyze the impact of the intervention group on students’ learning outcomes. We set up a two-level regression model with teachers on the higher level and students representing the lower level. All analyses were conducted in Mplus, version 6.11 (Muthén & Muthén, 1998–2010).

To produce an estimate of the intraclass correlation, we ran the *intercept-only* model, which contains no explanatory variables. The equation for this model reads *outcome*_*ij*_ = *γ*_*00*_ + *u*_*0j*_ + *e*_*ij*_, where *γ*_*00*_ is the overall intercept, and* u*_*0j*_ and *e*_*ij*_ are the residual errors at the teacher level and at the individual student level, respectively. The student index *i* runs from 1 to 604, and the teacher index *j* runs from 1 to 29. The outcome variables were the KCT as posttest and follow-up test and the test on quantitative problem solving.

The intercept-only models revealed intraclass correlations equal to 0.232, 0.228, and 0.267 for the outcomes in the KCT posttest, KCT follow-up test, and quantitative test (which was administered only as posttest), respectively. Thus, in all tests, considerably high amounts, i.e., 23 to 27%, of the variance of scores were at the class level where the intervention took place.

In addition to the intercept-only model without explanatory variables, we applied a more general model including KCT pretest scores (“KCTpre”), intelligence (“IQ”), and gender (“G”) as explanatory variables at the student level. At the higher level, we used the intervention conditions (“FA”, “FT”) as predictors for the three outcome variables. The active control TT group was defined as the baseline. We restricted our regression model to fixed slopes, and we excluded cross-level interactions to have appropriate statistical power. The mathematical equation representing this model is given by the following:

$$outcom{e}_{ij}={\gamma }_{00}+{\gamma }_{10}\bullet KCTpr{e}_{ij}+{\gamma }_{20}\bullet I{Q}_{ij}+{\gamma }_{30}\bullet {G}_{ij}+{\gamma }_{01}\bullet F{T}_{j}+{\gamma }_{02}\bullet F{A}_{j}+{u}_{0j}+{e}_{ij}$$

The variables *KCTpre* and *IQ* were grand mean-centered before analysis. The variable for gender (*G*: 0 = female, 1 = male) was centered at 0.5. The variable *FT*_*j*_ equaled one for teachers in the FT group and zero otherwise. Accordingly, *FA*_*j*_ was set to one only for teachers in the FA group and zero otherwise. The estimated intercept *γ*_*00*_ can be interpreted as the expected outcome for an average student, disregarding gender, with average intelligence and prior knowledge who underwent traditional teaching. The slopes *γ*_*01*_ and *γ*_*02*_ estimate the effect of frequent testing and formative assessment, respectively, on the outcomes after controlling for the predictors on the student level, represented by slopes *γ*_*10*_, *γ*_*20*_, and *γ*_*30*_. When evaluating the quantitative test scores as outcomes, slope *γ*_*10*_ was set to zero because we applied the KCT pretest particularly to measure gains in conceptual knowledge. To compare the outcomes of the FA and FT groups, we complemented our Mplus analyses with post hoc tests conducted in the R-software environment including the package *lme4* (R Development Core Team, 2013).

The estimates, standard errors, and two-tailed *p*-values for the more general model including explanatory variables on both levels are presented in Table 3.

**Table 3:** Nonstandardized estimates of the scores of the KCT posttest and follow-up and the quantitative test obtained from two-level regression

Variables	KCT posttest		KCT follow-up		Quantitative test	
	Estimate (SE)	*p*	Estimate (SE)	*p*	Estimate (SE)	*p*
Intercept *γ*_*00*_	24.53 (1.10)	< 0.001	25.67 (1.29)	< 0.001	18.91 (0.80)	< 0.001
Within level						
KCT pretest *γ*_*10*_^a^	0.644 (0.039)	< 0.001	0.670 (0.042)	< 0.001	-	
Intelligence *γ*_*20*_^a^	0.416 (0.057)	< 0.001	0.393 (0.072)	< 0.001	0.343 (0.080)	< 0.001
Gender *γ*_*30*_^b^	3.53 (0.57)	< 0.001	2.92 (0.65)	< 0.001	4.82 (0.66)	< 0.001
Residual variance of *e*	33.4 (2.3)	< 0.001	39.1 (4.3)	< 0.001	42.0 (3.0)	< 0.001
Between level						
Frequent testing *γ*_*01*_^c^	2.19 (1.27)	0.084	1.43 (1.44)	0.323	− 1.80 (1.60)	0.26
Formative assessment *γ*_*02*_^d^	5.48 (1.31)	< 0.001	4.62 (1.62)	< 0.001	− 2.31 (1.91)	0.23
Residual variance of *u*_*0*_	4.99 (2.60)	< 0.001	7.37 (2.99)	< 0.001	16.1 (4.7)	< 0.001
*R*-square						
Within level	0.589 (0.031)	< 0.001	0.553 (0.044)	< 0.001	0.163 (0.039)	< 0.001
Between level	0.501 (0.218)	0.021	0.335 (0.213)	< 0.001	0.056 (0.078)	0.478

Estimates on the between-level compared to traditional teaching

^a^KCT Pretest and Intelligence were grand mean centered. ^b^Gender was centered at 0.5 (0 = female, 1 = male). ^c^0 = traditional teaching, 1 = frequent testing. ^d^ 0 = traditional teaching, 1 = Formative assessment

Considering the KCT posttest scores as outcomes, the standardized regression slopes for the KCT pretest (0.59), intelligence (0.21), and gender (0.20, female = 0, male = 1) were all significant (*p* < 0.05) and positive. Obviously, prior knowledge affected KCT posttest scores even more than intelligence and gender. The three predictors used in the model explained approximately 60% of the variance on the student level. Regarding the quantitative test, the regression coefficients for intelligence and gender were also statistically significant, with a standardized value of 0.34 for gender and 0.22 for intelligence. In the next step, we investigated group differences and had a closer look to the interaction between group and gender.

### Between-group differences in conceptual understanding and problem-solving skills

In both the KCT posttest and follow-up test, the FA group significantly outperformed the FT group (posttest *d* = 0.37, follow-up *d* = 0.39) and the TT group (posttest *d* = 0.68, follow-up *d* = 0.62), all *p*s < 0.01. The FT group did not outperform the TT group in the posttest (*p* = 0.084) or the follow-up test (*p* = 0.323)*.* The comprehensive FA training was superior to the mere presentation of conceptual problems (FT), a condition which did not significantly improve conceptual understanding compared to traditional teaching.

For the quantitative test, no group differences in mean performance were found (all *p*s > 0.20). Classroom practice with a stronger focus on conceptual understanding was not at the expense of performance in quantitative problem solving.

**Fig. 6:**
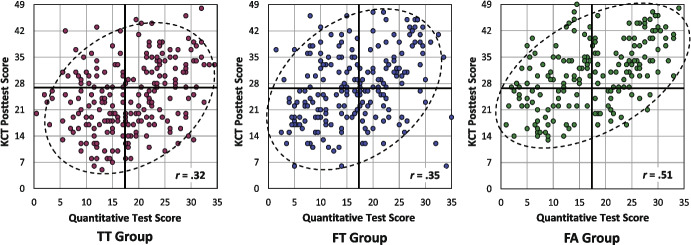
Scatter plots of the KCT posttest scores and quantitative test scores for the three groups. Each dot represents a student. The bold horizontal and vertical lines show the grand means of the test scores. The dashed ellipses represent 90% confidence regions of the covariance matrices. Correlations are displayed in the lower right corner of each diagram

Correlations between KCT and quantitative problem solving are depicted together with the scatterplots in Fig. 6. The higher the correlation the better a condition promoted an integrated understanding of kinematics.

The correlations for the three groups significantly differed from zero (all *p*s < 0.001). The correlation of *r* = 0.51 in the FA group was significantly higher than that in the TT group (*p* = 0.022) and considerably higher than that in the FT group (*p* = 0.058). This is illustrated in Fig. 6 by the shape of the ellipses, which represent 90% confidence regions of the covariance matrices (see Draper & Smith, 1981). The smaller the height of the ellipse compared to its width, the higher the correlation of the variables is. Figure 6 also shows that the proportion of students in the lower right area of the scatterplot is notably lower in the FA group (13%) than in the TT (27%) and FT groups (19%). The students in this area performed above average in the quantitative test but below average in the concept test. They successfully applied quantitative problem-solving strategies despite a limited understanding of the underlying kinematics concepts. We therefore confirmed that in classes where teachers had completed formative assessment training, students were likely prevented from using formulae without a deeper conceptual understanding.

### Gender differences

**Fig. 7:**
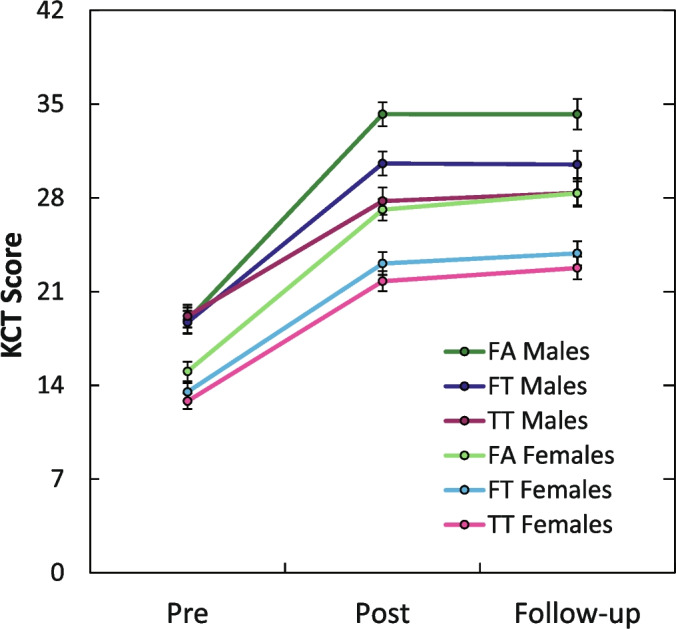
KCT scores separately presented for males and females. Mean group scores for the test on conceptual understanding, split by gender, are presented for three different time points: directly before the intervention (Pre), immediately following it (Post), and 3 months later (Follow-up). Error bars represent standard errors

Males outperformed females in all tests at all measurement points, as the regression coefficients in Table 3 suggest. Figure 7 depicts the scores in the KCT separately for males and females. Post hoc tests showed that male FA students outperformed all other groups (*p* < 0.05) in the posttest. The gender gap at the posttest level expressed in effect sizes was *d* = 0.87 for the FA group, *d* = 0.84 for the FT group, and *d* = 0.67 for the TT group. Thus, formative assessment did not enable female students to catch up with their male peers. Nonetheless, female students did benefit from formative assessment. Post hoc tests revealed that female FA students and male FT and TT students outperformed female students from the TT and FT groups on the posttest (all *p*s < 0.03). Female students in the FA group were able to catch up with male students in the TT and FT groups. However, this only holds for conceptual understanding. In the quantitative test, the average scores for males were *M* = 21.6 (*SD* = 6.4), *M* = 18.1 (*SD* = 8.0), and *M* = 20.5 (*SD* = 7.9) in the TT, FT, and FA groups, respectively, while the corresponding average scores for females were *M* = 15.8 (*SD* = 7.5), *M* = 14.6 (*SD* = 7.2), and *M* = 14.5 (*SD* = 8.6). A significant gap (*p* < 0.001) between female FA students and male TT students still existed in the quantitative test.

## Discussion

Formative assessment is a widely accepted approach to supporting meaningful and sustainable learning. In accordance with the constructivist view of learning, formative assessment aims to diagnose prior knowledge and to support learners in aligning it with scientifically recognized knowledge. However, while the usefulness of formative assessment in promoting conceptual understanding is evident, even for teachers who agree with its mission, effectively putting it into practice remains a challenge. On one hand, designing or selecting appropriate concept questions and integrating them within the key strategies of formative assessment places high demands on teachers. We therefore investigated whether the implementation of these key strategies is more beneficial than merely applying concept questions, especially for above-average achieving students. On the other hand, implementing formative assessment requires time, which may come at the expense of promoting procedural knowledge. This is particularly important in content areas where competence is reflected in an integration of conceptual understanding and mastering procedures, as it is the case in mathematics or physics. For elementary and early secondary school mathematics, a potential conflict in doing justice to both competencies has already been recognized (Andersson & Palm, 2017; Clarke et al., 2014). We investigated whether this conflict in time allocation also appears in the area of kinematics with more advanced learners. We therefore compared the effects of an intervention where teachers were provided only with conceptual questions (FT group) or taught kinematics in a traditional way (TT group) against the effects of a comprehensive formative assessment program (FA group) based on the five strategies defined by Black and Wiliam (2009). By implementing technology-based tools, teachers got the opportunity to diagnose and monitor their students’ pitfalls and progress and react accordingly, and learners got detailed information about the profile of their knowledge, and what they need to work on.

We showed that a 1-day training on using tools of formative assessment enabled teachers to provide learning environments in kinematics that significantly improved the conceptual understanding of 15-year-old students. These students outperformed those who underwent traditional teaching (TT) and those who underwent traditional teaching supplemented by the frequent application of concept questions (FT), in line with our first hypothesis. However, there was no significant difference between students in the TT and FT groups, contrary to our second hypothesis. We could thereby show that the mere presentation of conceptual problems was not sufficient for promoting meaningful learning even in our sample of above-average students. Our results confirm the benefits of formative assessment in classes with above-average students and teachers who have a strong background in physics.

The comprehensive formative assessment program did not benefit conceptual understanding at the expense of procedural competencies. No differences between the groups were found with regard to the quantitative problem-solving test which assessed the equally important competence of mathematically modeling motions. This result must be seen against the background that quantitative problem solving is a central component of traditional teaching. Although the students in the FA group spent less time solving quantitative problems than did the students in the TT and FT groups, they did not perform significantly worse on these problems on average. This finding supports our expectation that less practice in quantitative problem solving is necessary if the students have a better conceptual understanding, consistent with hypothesis 3a. The results are in line with the findings from many other studies that have shown an indirect effect of conceptual understanding on quantitative problem solving in physics (Crouch & Mazur, 2001; Hofer et al., 2018; McDaniel et al., 2016; Thacker et al., 1994). The considerable correlation of *r* = 0.51 found in the FA group between the performance in the conceptual and in the quantitative test suggests that students from FA classes were able to better integrate concepts and quantitative strategies, supporting hypothesis 3b. In the FA group, there were fewer students with poor conceptual understanding but an above-average achievement in quantitative problem solving than in the other two groups. Especially in the TT group, a substantial number of students performed better on the quantitative test than on the concept test. For these students, it was obviously possible to apply memorized algorithms without linking them to underlying physics concepts.

Concerning the gender gap, our results are in line with numerous findings in the STEM area: males outperformed females in all groups. There was only partial confirmation that formative assessment could be a lever to change this outcome. Male students benefited the most from formative assessment, which widened rather than narrowed the gender gap. Nonetheless, the females in the FA group outperformed the females in the FT and TT groups and caught up with the males in the FT and TT groups, which indicates that formative assessment is among the means of classroom practice that support female students’ conceptual understanding.

### Limitations of our study and open questions for future research

By running a cluster-randomized field study and by considering the hierarchical structure of our data in the statistical analysis, we fulfilled the methodological standards for intervention classroom studies. However, as the teachers of our study participated on a voluntary basis, they entered the study with a positive attitude towards adapting their classroom practice. The questionnaire on pedagogical content knowledge reflected the acceptance of the constructivist view of learning. It is, however, quite likely that teachers differ in the extent to which they accept the mission behind formative assessment and the extent to which they are able to implement formative assessment appropriately in their classroom. Identifying teacher characteristics that contribute to the success of formative assessment should be a focus of future research.

In addition, more frequent and more fine-grained classroom observations than we could realize in our study may help to identify and overcome potential shortcomings of teacher training. While we demonstrated that teachers can implement training on formative assessment to enhance students’ conceptual understanding, it remains uncertain whether those who underwent the training will apply it in their future classes on kinematics and beyond. Future research needs to explore whether these trained teachers will adjust their teaching practice when covering other topics.

In this study, formative assessment focused on fostering conceptual understanding. However, following a constructivist perspective, formative assessment could also involve diagnosing, providing feedback, and offering targeted support for the construction of other types of knowledge. Future studies could aim to identify characteristics of teacher training that enable teachers to apply formative assessment flexibly to support the construction of different types of knowledge.

## Supplementary Information

Below is the link to the electronic supplementary material.

English translation of the quantitative problem-solving test including the assignment of problems to the kinematics concepts, the solutions, and the rating grid (PDF 1 MB)

## Data Availability

The data that support the findings of this study are available from the corresponding author upon reasonable request.
