# Supplementary material for: Enhanced conceptual understanding through formative assessment: results of a randomized controlled intervention study in physics classes
Source: Educ Assess Eval Account. 2024 Dec 27;37(1):5–33. doi: 10.1007/s11092-024-09445-6 (PMC11958380; doi:10.1007/s11092-024-09445-6)

Kinematics Test on Quantitative Problem Solving

An English translation of the conventional test problems used in the study is provided on the following pages. The problems are based on the following seven kinematics concepts:

C1: Velocity as rate
C2: Velocity as 1D vector
C3: Addition of velocities in 2D
C4: Displacement as area under the *v,t*-curve
C5: Acceleration as rate
C6: Acceleration as 1D vector
C7: Velocity change as area under the *a,t*-curve

The problems of the test can be assigned to the kinematics concepts according to the following table.

Problem	Subproblem	Applied concept	Concept covered by formulas	Additional tasks
1	a	C5		
	b		C7	
2	a		C4, C7	
	b	C1		
3	a	C3		
	b	C1		
4	a			Sketch curves in the *x,t*-diagram
	b			Read out from *x,t*-diagram
	c		C4, C7	
5	a	C1	C4, C7	
	b			Sketch curve in the *v,t*-diagram

Note

We believe that students do not apply these concepts in every case. For example, in problem 2a the displacement has to be computed for a given acceleration and initial velocity. Thus, concepts C4 and C7 are required. But we assume that students rather try to find the solution using the formulas derived during instruction than apply C4 and C7.

TEST ON QUANTITATIVE PROBLEM SOLVING IN

K I N E M A T I C S

Nickname	
SuS-Code	

Consider the following:

- Fill in your nickname and your student number.
- Test time: 45 min.
- Formula booklet and calculator are allowed.
- On each problem, please write the formula you have used and the numbers you have plugged in. If you don't do so, you won't get full credit.
- The solution can be written with any correct unit (e.g., the velocity either in m/s or in km/h).
- In curly brackets you can find reasonable values of the previous subproblem so that you can continue with the solution of the next problem. The given values do not, however, represent the correct result of the previous subproblem.
- Good luck!

Problem 1 (4P)

An airplane is landing at the airport in Zurich and touches down on the runway. Before the pilot starts to brake, the airplane has a velocity of 75 m/s. After 18 s the airplane comes to rest.

a. What is the average acceleration of the airplane during the braking process?

$$a = \frac{v_f - v_i}{\Delta t} = \frac{0 - 75\,\frac{\mathrm{m}}{\mathrm{s}}}{18\ \mathrm{s}} = -4.2\ \frac{\mathrm{m}}{\mathrm{s}^2} \qquad \text{1P + 1P}$$

b. What is the speed of the airplane 6.0 s after the pilot begins to brake?

$$v(6\ \mathrm{s}) = v_i + a \cdot t = 75\ \frac{\mathrm{m}}{\mathrm{s}} - 4.2\ \frac{\mathrm{m}}{\mathrm{s}^2} \cdot 6.0\ \mathrm{s} = 50\ \frac{\mathrm{m}}{\mathrm{s}} \qquad \text{1P + 1P}$$

Problem 2 (8P)

A car drives with a speed of 16 m/s on a straight road. Suddenly in front of it a motorcycle appears from a side road. Immediately (no reaction time) the car driver hits the brake and decelerates the car with -3.2 m/s^2.

a. How far does the car move before it stops?

{If you cannot solve this problem assume that the car moves 38 m before it stops, in order to solve b.}

$$v = v_0 + at \ \rightarrow\ t = \frac{v - v_0}{a} = \frac{0 - 16\,\frac{\mathrm{m}}{\mathrm{s}}}{-3.2\,\frac{\mathrm{m}}{\mathrm{s}^2}} = 5.0\ \mathrm{s} \qquad \text{1P + 1P}$$

$$x = x_0 + v_0 t + \frac{1}{2} a t^2 = 0 + 16\frac{\mathrm{m}}{\mathrm{s}} \cdot 5.0\ \mathrm{s} - \frac{1}{2} 3.2\ \frac{\mathrm{m}}{\mathrm{s}^2} \cdot (5.0\ \mathrm{s})^2 = 40\ \mathrm{m} \qquad \text{1P + 1P + 1P}$$

or:

$$x = \frac{v_f^2 - v_i^2}{2a} = 40\ \mathrm{m} \qquad \text{5P}$$

b. Assume that the initial distance between the motorcycle from the sideroad and the car is 55 m. What would be the maximal reaction time of the car driver, so that the car stops right in front of the motorcycle?

$$\Delta t = \frac{\Delta x}{v_0} = \frac{55\ \mathrm{m} - 40\ \mathrm{m}}{16\,\frac{\mathrm{m}}{\mathrm{s}}} = 0.94\ \mathrm{s} \qquad \text{1P + 1P + 1P}$$

Problem 3 (5P)

At the airshow 2014 in Payerne, two small airplanes are flying an aerobatic manoeuvre. The two airplanes fly in a plane parallel to the surface of the Earth on perpendicular flight paths. The first airplane has a speed of 180 km/h and the second has a speed of 140 km/h. The two airplanes are 500 m apart from each other on a collision course.

a. With what speed does the pilot in one airplane see the other airplane approaching?

{If you cannot solve this problem use 200 km/h for the speed to continue the problem.}

$$v = \sqrt{v_1^2 + v_2^2} = \sqrt{\left(180\ \frac{\text{km}}{\text{h}}\right)^2 + \left(140\ \frac{\text{km}}{\text{h}}\right)^2} = 228\ \frac{\text{km}}{\text{h}}$$

1P (Sketch) + 1P + 1P

b. Thanks to the CAS (collision avoiding system) the collision could be prevented. However, what would have been the time before the two airplanes collided?

$$v = 228\ \frac{\text{km}}{\text{h}} = 63.3\ \frac{\text{m}}{\text{s}}$$

$$\Delta t = \frac{\Delta x}{v} = \frac{500\ \text{m}}{63.3\ \frac{\text{m}}{\text{s}}} = 7.89\ \text{s}$$

1P + 1P

Problem 4 (7P)

Luca drives with his motorcycle to the school. His bag on the carrier gets loose and falls to the ground. He stops and while he fixes the bag again on the carrier he sees at time point $t = 0$ his classmate Celine at a distance of 170 m in front of him. She drives with constant speed of 4 m/s towards Luca. After 5 s ($t = 5$ s) Luca is ready to continue his ride. He drives with an acceleration of 0.8 m/s^2 towards Celine.

a. Sketch the rides of Celine and Luca in the x,t-diagram below.

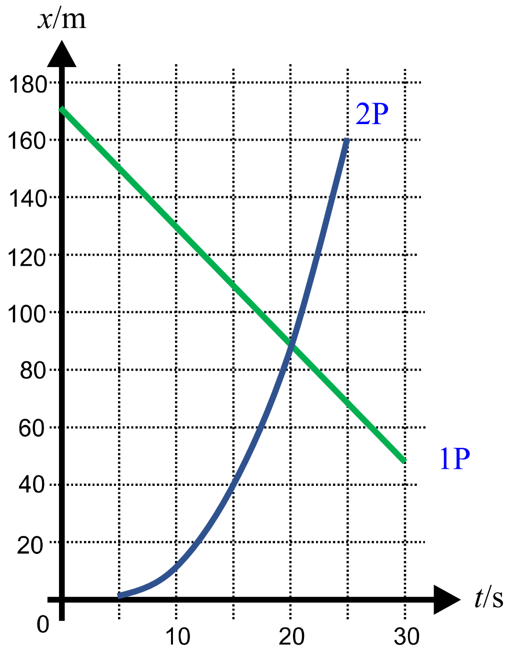

b. Read off the diagram the approximate meeting time t^* of Celine and Luca.

$t^* = 20$ s. 1P

c. Write an equation in order to determine the meeting time t^*.

Celine: $x_C = x_0 - v_C t^* = 170\text{ m} - 4\frac{m}{s}t^*$ 1P

Luca: $x_L = \frac{1}{2}a(t^* - t_0)^2 = \frac{1}{2}0.8\ \frac{\text{m}}{\text{s}^2}(t^* - 5\text{ s})^2$ 1P

Meeting point: $x_C = x_L$

$170\text{ m} - 4\ \frac{\text{m}}{\text{s}}t^* = \frac{1}{2}0.8\ \frac{\text{m}}{\text{s}^2}(t^* - 5\text{ s})^2$ 1P

Problem 5 (11P)

Because of safety reasons, an Intercity train moving with constant speed of 144 km/h has to slow down when passing a local train station without stopping. For a distance of 800 m the speed is reduced to 57.6 km/h. The train slows down with 0.80 m/s^2 and increases speed with 0.60 m/s^2.

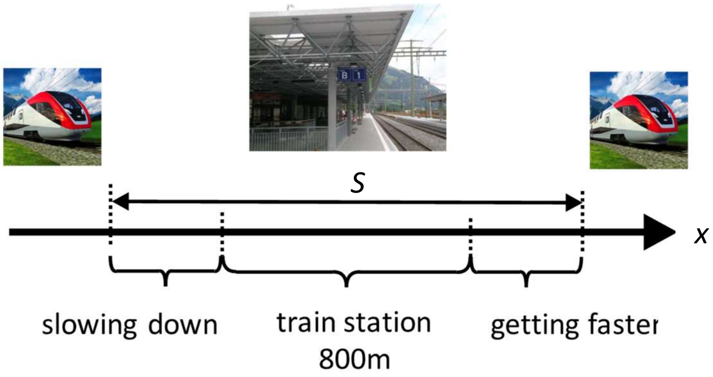

a. For how long does the train move with reduced speed?

$144\ \frac{\text{km}}{\text{h}} = 40\ \frac{\text{m}}{\text{s}};\ 57.6\ \frac{\text{km}}{\text{h}} = 16\ \frac{\text{m}}{\text{s}}$ 1P

Slowing down: $v = v_0 + at \rightarrow \Delta t_1 = \frac{v - v_0}{a} = \frac{16\frac{\text{m}}{\text{s}} - 40\frac{\text{m}}{\text{s}}}{-0.80\frac{\text{m}}{\text{s}^2}} = 30\ \text{s}$ 1P + 1P

Station: $\Delta t_2 = \frac{\Delta x}{v} = \frac{800\ \text{m}}{16\frac{\text{m}}{\text{s}}} = 50\ \text{s}$ 1P + 1P

Getting faster: $v = v_0 + at \rightarrow \Delta t_3 = \frac{v_f - v}{a} = \frac{40\frac{\text{m}}{\text{s}} - 16\frac{\text{m}}{\text{s}}}{0.60\frac{\text{m}}{\text{s}^2}} = 40\ \text{s}$ 1P + 1P

Total $\Delta t_{tot} = \Delta t_1 + \Delta t_2 + \Delta t_3 = 30\ \text{s} + 50\ \text{s} + 40\ \text{s} = 120\ \text{s}$ 1P

b. Sketch the *v*,*t*-diagram of the passage of the train through the station for the distance *S*.

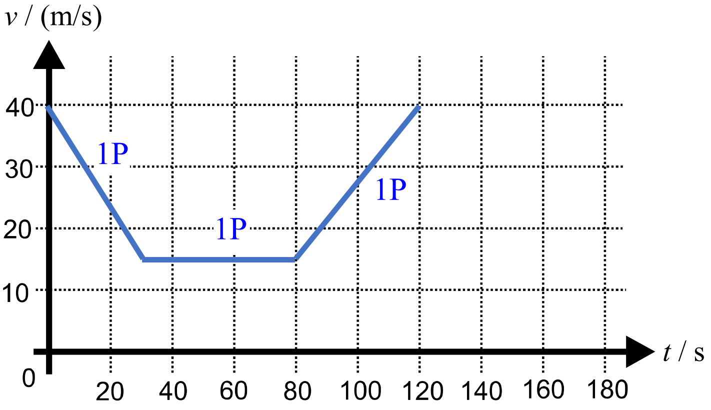

Supplement: Supplementary file 1 — English translation of the quantitative problem-solving test including the assignment of problems to the kinematics concepts, the solutions, and the rating grid (PDF 1 MB) [file 11092_2024_9445_MOESM1_ESM.pdf]
